# The Habenula’s role in major depressive disorder: recent insights from preclinical and human studies

**DOI:** 10.1038/s41398-026-03867-0

**Published:** 2026-02-07

**Authors:** Feiteng Lin, Kayleigh Casmey, Sierra A. Codeluppi-Arrowsmith, Gustavo Turecki

**Affiliations:** 1 Department of Human Genetics, McGill University, Montréal, Canada; 2 Integrated Program in Neuroscience, McGill University, Montréal, QC Canada; 3 McGill Group for Suicide Studies, Douglas Institute, Department of Psychiatry, McGill University, Montréal, QC Canada

**Keywords:** Depression, Molecular neuroscience

## Abstract

The habenula is a small epithalamic structure composed of two distinct subregions, the medial (MHb) and lateral (LHb) habenula. It serves as a critical hub for integrating fronto-limbic and brainstem signals to regulate motivation, mood, and reward processing. Therefore, it is unsurprising that dysfunction of the habenula has been implicated in several mood disorders including major depressive disorder (MDD), a debilitating mood disorder marked by low mood and feelings of hopelessness. This review synthesizes recent advances in understanding the habenula’s neurocircuitry, molecular landscape, and role in MDD pathophysiology, while evaluating its potential as a therapeutic target. Specifically, emerging evidence highlights subregion-specific pathology. Indeed, in MDD and in animal models of depression, the MHb has been shown to exhibit marked downregulation of calcium-dependent activator protein for secretion 2 (CAPS2) and deficits in nicotinic acetylcholine receptor-mediated signaling. While in the LHb, dysregulated expression profiles of inward-rectifying potassium channel Kir4.1, the β isoform of calcium/calmodulin-dependent protein kinase II (CaMKIIβ), protein phosphatase 2 A (PP2A), and small nucleolar RNA SNORA69 have been found in animal models of depression and MDD postmortem studies. Structural imaging and postmortem neurohistological studies in MDD patients have further revealed habenular volume changes, reduced neuronal cell counts, diminished cell area, and abnormal functional connectivity. As research unravels the habenula’s complexities, its potential in treating mood disorders grows increasingly salient, offering new avenues for intervention in mental health.

## Introduction

### Structural and functional organization of the habenula

**Fig. 1:**
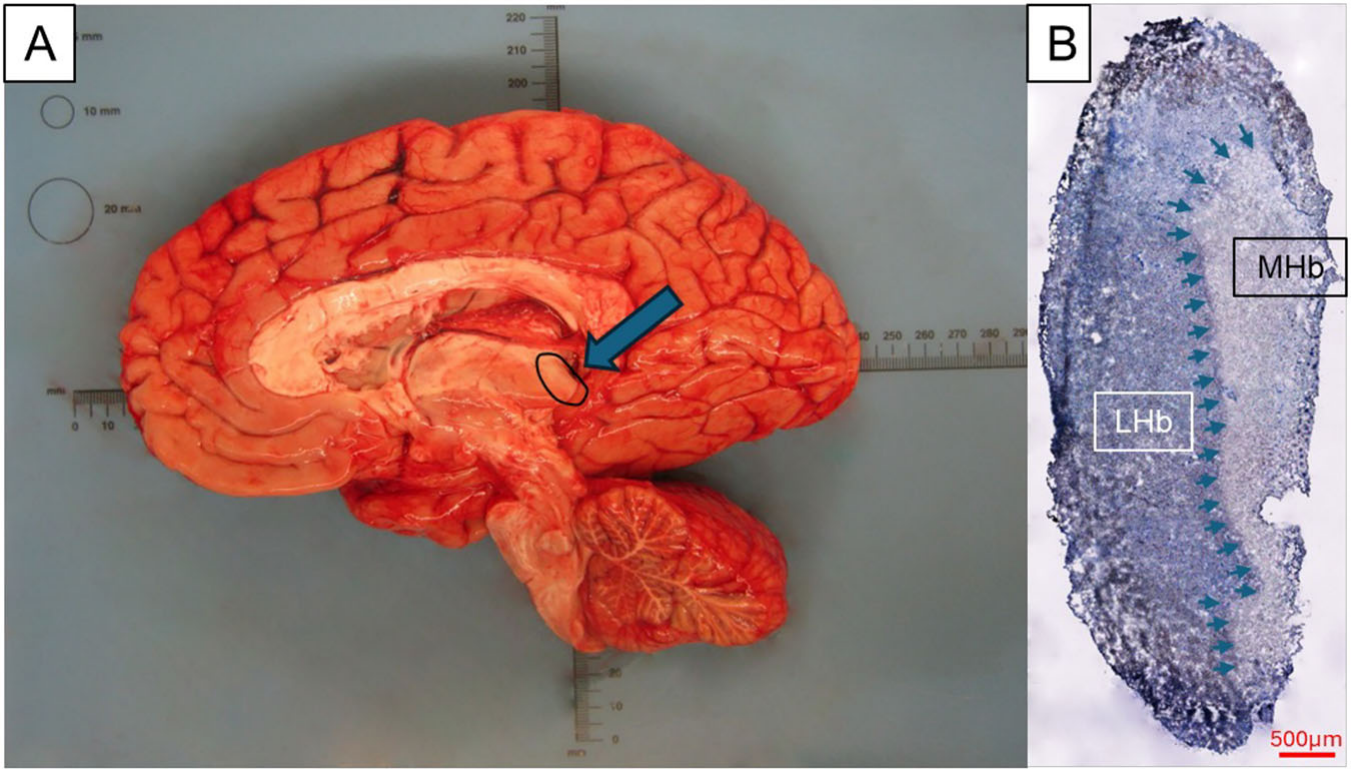
Location of the human habenula (Hb) and the subdivision of the medial habenula (MHb) and lateral habenula (LHb). **A** Image of the right hemisphere of the human brain cut sagittalyl along the midline with a black circle and a blue arrow indicating the location of the right Hb. **B** Woelcke staining of a habenula slice, with blue arrows indicating the border of the LHb and the MHb based on different myelinated fibers within the subregions.

The habenula is a small epithalamic structure composed of two nuclei in each hemisphere (Fig. 1A) that plays a crucial role in modulating motivation, emotion, and reward-related processes [1–4]. The left and right habenula nuclei are connected by the habenular commissure, a white matter tract located on either side of the midline [5]. Notably, the habenula exhibits high myelin content, consisting of multiple white matter tracts and intrinsic fibers due to its extensive interconnectivity with other brain regions [6–9]. The habenula is subdivided into two subregions the medial (MHb) and lateral (LHb), making up 9 and 91% of the human habenula, respectively [6]. These subregions are characterized by distinct neuronal populations and differing myelin content, which we briefly outline below (Fig. 1B) [10–13].

**Fig. 2:**
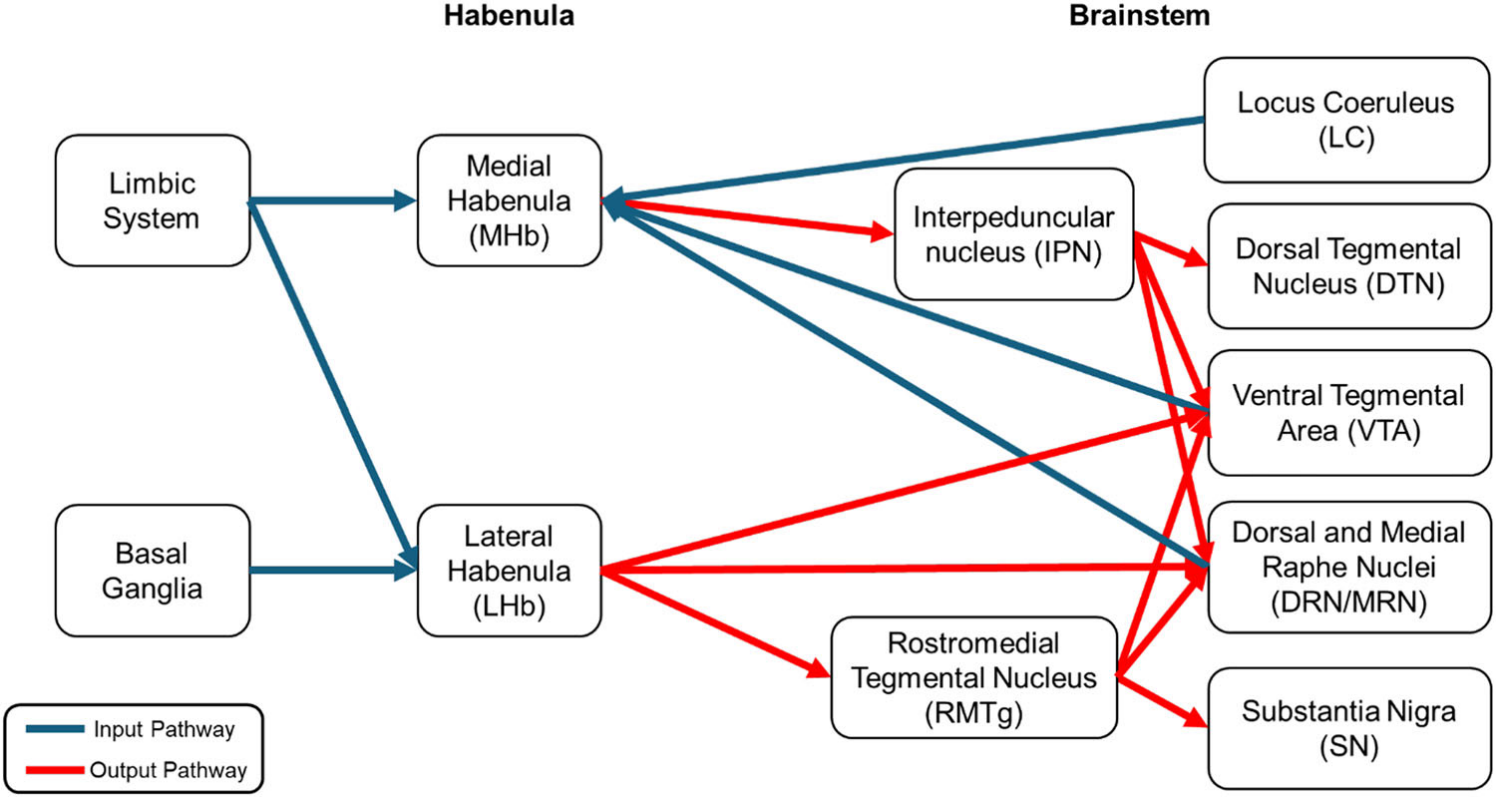
Input and output connectivity pathways of the habenula to other regions. The MHb mainly receives signals from the septum within the limbic system and relays information downstream to the brainstem through the IPN. The LHb receives input from the limbic system and basal ganglia, regulating neurotransmission to the brainstem. Through glutamatergic projections, the LHb activates GABAergic neurons in the RMTg that inhibit dopaminergic cells in the SNc and VTA, while also sending direct inputs to the VTA and DRN/MRN.

The MHb is primarily comprised of cholinergic and substance P-ergic neurons, characterized by cells that are mainly small, round, and heavily packed together [6, 14–16]. As illustrated in Fig. 2, the MHb primarily receives cholinergic and gamma-aminobutyric acid (GABA)-ergic inputs from the medial septum and the diagonal band of Broca, as well as dopaminergic input from the ventral tegmental area (VTA), and noradrenergic inputs from the locus coeruleus, and the superior cervical ganglion [17, 18]. The interpeduncular nucleus (IPN), a structure positioned along the ventral midline of the midbrain, receives output from the MHb [19, 20] via the projection of the fasciculus retroflexus [21]. The IPN projects to brainstem regions that regulate neurotransmitter release, including the dorsal tegmental nucleus [19, 22], the VTA [20], and both dorsal and medial raphe nuclei [18, 20, 23]. The intricate connectivity of the MHb along with its influence on various neurotransmitter systems through the IPN, makes it a key modulator of brainstem activity. Indeed, the MHb conveys the information from the upper brain regions by influencing monoaminergic neurotransmitter release through its projection to the brainstem.

The LHb is predominantly composed of glutamatergic neurons expressing vesicular glutamate transporters (VGlut) 2 and 3 [24–26], distinguishing it as a primarily excitatory structure. It is further characterized by a large degree of variability in the size and shape of cells, and loose packing as compared to the MHb [6, 27]. Similarly to the MHb, it functions as a central communication hub, conveying emotional information from both the limbic system and the basal ganglia, further exerting influence on neuromodulatory systems (Fig. 2) [28]. The LHb primarily projects to several key brainstem regions involved in neuromodulation, including the VTA and substantia nigra pars compacta (SNc) which are associated with dopaminergic signaling, the dorsal and medial raphe nuclei, which contribute to serotonergic regulation, and the laterodorsal tegmental nucleus, which plays a role in cholinergic pathways [21, 29, 30]. The LHb not only forms direct glutamatergic connections with the VTA [31], but also sends glutamatergic projections to the rostromedial tegmental nucleus (RMTg), whose GABAergic neurons inhibit dopaminergic neurons in the SNc and VTA [2, 32, 33]. Moreover, output from the LHb exerts rapid negative regulation on dopaminergic and serotonergic neurons, resulting in an intermediate and potent inhibitory response in these neuronal populations [3, 34–40].

### Habenular implication in MDD

Major depressive disorder (MDD) is the leading cause of global disease burden and disability [41]. Its clinical presentation is marked by persistent low mood, anhedonia, appetite changes, psychomotor alterations, sleep disturbances, and suicidal thoughts and behaviors [42]. While longstanding theories have attributed MDD to a monoamine imbalance [43–45], emerging evidence now suggests that these monoaminergic changes may be downstream effects of dysregulated neural circuits, particularly involving the habenula, which plays a central role in the reward system and the regulation of mood [46–48]. Indeed, the habenula is known to modulate behavior by integrating aversive and reward-related signals to regulate key neurotransmitter systems in the brainstem [3] making its implication in MDD evident. In this review, we will highlight both preclinical and clinical studies which consistently associate habenular dysfunction with MDD, underscoring its significance in the disorder’s complex neurobiology.

## Habenular dysfunction in depressive-like behaviour: insights from animal models

Animal models are indispensable tools for elucidating the biological underpinnings of disease, including mood disorders such as MDD [49]. Various strategies for modeling the pathology of MDD in rodents have been employed such as stress paradigms with have high face and predictive validity [49–52]. Stress is highly comorbid with MDD and has been shown to be a key environmental factor [53]. Rodent stress paradigms are widely used, including unpredictable chronic mild stress (UCMS) [54, 55], chronic restraint stress (CRS) [53, 56], social defeat (SD) [57], and learned helplessness (LH) [58, 59]. Animal models can be used to target and manipulate specific regions, subtypes of cells and proteins in the brain to determine casual effects of various molecular manipulations [51, 60, 61]. Emerging research in rodent models of stress-induced depressive-like symptoms highlights the essential role of the habenula, revealing distinct molecular alterations in its medial and lateral subregions across various stress paradigms. In the following subsections, we detail how these findings in animal models illuminate the habenula’s role in depression and may inform novel therapeutic strategies (Table 1).

**Table 1:** Molecular Expression and Circuit Alteration in the Habenula.

Region	Molecule(s)	States in Rodent Models of Depressive-like Symptoms/Individual with MDD	Citations
Medial Habenula (MHb)	**CAPS2**	Downregulated (*n* = 8; 50% CRS rodent model) (*n* = 23; ~52.2% post-mortem samples from patients with MDD)	*Down-regulation of habenular calcium-dependent secretion activator 2 induces despair-like behavior* [62]
	**Cholinergic signaling genes (CHAT, VACHT, CHT, CHRNA3, CHRNB3, and CHRNB4)**	Downregulated (*n* = 8; 50% CRS rodent model) (*n* = 23; ~52.2% post-mortem samples from patients with MDD)	*Down-regulation of cholinergic signaling in the habenula induces anhedonia-like behavior* [66]
Lateral Habenula (LHb)	**Kir4.1**	Upregulated (*n* = 14; ~64.3% LH rodent model)	*Astroglial Kir4.1 in the lateral habenula drives neuronal bursts in depression* [69]
	**βCaMKII**	Upregulated (*n* = 15; ~46.7% CRS rodent model)	*βCaMKII in Lateral Habenula Mediates Core Symptoms of Depression* [72]
	**p11**	Upregulated (*n* = 16; ~62.5% LH rodent model)	*Elevation of p11 in lateral habenula mediates depression-like behavior* [76]
	**SNORA69**	Upregulated (*n* = 15; ~53.3% UCMS rodent models) (*n* = 30; 50% post-mortem samples from patients with MDD)	*SNORA69 is up-regulated in the lateral habenula of individuals with MDD* [84]
	**ErbB4**	Downregulated (*n* = 37; ~64.9% post-mortem samples from patients with MDD)	*miR-323a regulates ERBB4 and is involved in depression* [87]
	**REM Sleep-Active Neuron**	Increased Activities (*n* = 30; ~53.3% CRS rodent model)	*A potentiation of REM sleep-active neurons in the lateral habenula may be responsible for the sleep disturbance in depression* [78]
	**Stress Responsive Neurons**	Increased Activities (*n* = 16; 25% restraint stress, 25% feet shock, and 25% social defeat)	*A small population of stress-responsive neurons in the hypothalamus-habenula circuit mediates development of depression-like behavior in mice* [79]
	**Neuronal TCF7L2**	Downregulation (*n* = 28; 50% CUMS rodent model)	*Neuronal TCF7L2 in Lateral Habenula Is Involved in Stress-Induced Depression* [80]

*CAPS2* calcium-dependent activator protein for secretion 2, Kir4.1, Inwardly rectifying potassium channel subunit 4.1; βCaMKII, the β isoform of Ca^2+^/Calmodulin-dependent Protein Kinase II; *MDD* major depressive disorder; ErbB4, Erb-B2 receptor tyrosine kinase 4; TCF7L2, Transcription Factor 7-Like 2.

### The association of molecular alterations in the mhb with depressive-like behavior

Recent work investigating the MHb in CRS rodent models have revealed a significant reduction in CAPS2 mRNA in substance P-ergic, cholinergic, and glutamatergic neurons using quantitative PCR (qPCR) [62]. CAPS2 is critical for neuronal growth and function, facilitating the secretion of brain-derived neurotrophic factor [63]. In the MHb, CAPS2 is expressed in both dorsal and ventral regions, colocalizing with glutamatergic and cholinergic neurons [62, 64, 65]. Selective knock down of CAPS2 in the MHb using an adeno-associated virus 2/9 (AAV2/9) led to increased immobility in tail suspension test (TST) and forced swim test (FST), indicating enhanced despair-like behavior [62].

In addition, reduced MHb mRNA expression (qPCR) of key cholinergic signalling genes (CHAT, VACHT, CHT), and nicotinic receptor subunits (CHRNA3, CHRNB3, and CHRNB4) were found in the MHb of CRS models [66]. These cholinergic signalling genes, specifically CHAT, are essential for acetylcholine synthesis and release at the MHb–IPN synapses [67]. Interestingly, a habenula specific knockdown of CHAT via an AAV2/9 vector significantly reduced sucrose preference (SP) indicating that habenular CHAT plays a necessary role in the modulation of anhedonia-like behaviour [66]. These findings, together with pharmacogenetic studies showing that activation of habenular cholinergic neurons excites VTA dopamine neurons and suppresses DRN serotonergic activity, underscore that impaired habenular cholinergic signaling initiates a cascade of neurochemical changes culminating in depressive-like symptoms.

### The association of molecular alterations in the LHb with depressive-like behavior

The LHb exhibits a range of stress-induced molecular changes that vary by stress paradigms. Firstly, the inwardly rectifying potassium channel subunit 4.1 (Kir4.1), mainly found in astrocytes for spatial potassium buffering [68], is markedly upregulated at the mRNA and protein level in the LHb of rodents exposed to LH stress paradigm [69]. Furthermore, this study showed that an astrocyte-specific overexpression of Kir4.1 in the LHb increased neuronal bursting, and was sufficient to induce depressive-like behaviors in the FST and SP [69]. Conversely, Kir4.1 knockdown in LH rodents oppositely abolished increased bursting, and alleviated depressive-like symptoms [69].

Furthermore, the protein and mRNA expression of the β form of calcium/calmodulin-dependent protein kinase type II (βCaMKII), a serine/threonine kinase key to long-term potentiation and neuronal plasticity [70], is also upregulated in the LHb of LH rats [71–73]. Comparable observations were found in mice exposed to UCMS [72]. AAV2-mediated overexpression of βCaMKII in the LHb enhanced synaptic efficacy and induced depressive-like behaviors, while a targeted knockdown alleviated these symptoms [72].

Similarly, blocking protein phosphatase 2 A (PP2A) activity in the LHb helped restore normal electrical activity, as evidenced from the electrophysiological recording, and alleviated the depressive-like symptoms in the LH rodent model [74]. Under normal conditions, GABA_B_ receptor (GABA_B_R) activation triggers an outward potassium current via GIRK channels, suppressing neuronal excitability [75]. However, stress-induced PP2A hyperactivity dephosphorylates Ser783 on the GABA_B_2 subunit, leading to internalization of GABA_B_Rs and GIRK channels, thereby diminishing the inhibitory current and causing neuronal hyperactivity [74]. Pharmacological inhibition of PP2A restored GABA_B_-GIRK function and normalized the electrophysiological properties of LHb neurons in vivo, thereby rescuing depressive-like behaviors such as immobility in the FST, escape performance in the LH paradigm, and SP [74].

In addition, p11, an S100 EF-hand calcium-binding protein, has been shown to modulate synaptic and neuronal activity in the LHb via interactions with 5-HT receptors, ion channels, and chromatin modifiers linked to depression [76, 77]. Interestingly, p11 was found to be co-expressed with D2 receptor-containing glutamatergic LHb neurons [76]. In CRS rodent models, p11 protein expression, especially in the medial LHb, increased alongside elevated c-fos levels (a proxy of increased cellular activity), correlating with depressive-like behaviors that lasted up to 30 days [76]. A LHb-specific p11 knockdown in stressed mice normalized both neuronal excitability, the frequency of spontaneous inhibitory postsynaptic currents, and prevented depressive-like phenotypes [76]. Mice with overexpressed p11 in dopamine D2 receptor-containing glutamatergic LHb neurons of D2-Cre mice via an Cre-dependent viruses showed pronounced depressive- and anhedonia-like behaviors [76]. This data suggests that p11 in the LHb modulates inhibitory synaptic transmission and mood-regulating circuits of depressive-like behaviours [76].

More recent work has also significantly implicated LHb dysfunction in several rodent stress models. Indeed, a markedly increased proportion of REM sleep-active neurons was found in rodents exposed to CRS, along with enhanced burst firing activity [78]. When these neurons were selectively activated, it increased REM sleep duration and induced depressive- and anhedonia-like phenotypes, without affecting overall locomotion [78]. Conversely, targeted inhibition of REM sleep-active LHb neurons shifted their firing from burst to tonic mode, normalized REM sleep parameters, and alleviated depressive-like behaviors [78]. Additional recent studies of rodents exposed to CRS unveiled a similar increase in neuronal activity in the medial LHb and the middle lateral hypothalamus (mLH) [79]. When modulated, these subpopulation of neurons were also found to attenuate the depressive-like phenotypes outlined above [78, 79]. Additional work performing fluorescence staining of transcription factor 7-like 2 (TCF7L2) levels in the LHb showed significantly reduced levels in rodents exposed to CMS [80]. TCF7L2 is known to be a pivotal transcription factor in the Wnt signaling pathway which greatly influences diverse neuropsychiatric processes [81–83]. Notably, a LHb neuron-specific AAV-mediated knockdown of TCF7L2 actually elicited robust antidepressant-like effects, while conversely, overexpression of TCF7L2 induced marked depressive-like behaviors [80]. Notably, the administration of an N-methyl-D-aspartate receptor (NMDAR) agonist reversed the antidepressant effects observed with TCF7L2 knockdown, whereas treatment with an NMDAR antagonist alleviated the depressive phenotype driven by TCF7L2 overexpression [80]. This introduces the NMDA-pathway as a potential mechanism through which the habenula may be targeted for antidepressant treatment, discussed later in the text.

Additional studies in rodents exposed to UCMS found an upregulation of SNORA69 in the LHb that correlates with depressive-like behaviours [84]. SNORA69, is a small nucleolar RNA (snoRNA) guiding pseudouridylation of 5.8S rRNA and 18S rRNA in the human LHb, modifications essential for ribosomal function and translation fidelity [84, 85]. Elevated SNORA69 increases pseudouridylation at target rRNA sites, potentially disrupting ribosome-mediated translation of proteins critical for synaptic plasticity, monoaminergic signaling, and stress adaptation—pathways central to LHb function [84, 86]. Unlike antidepressant-responsive pathways, SNORA69 expression remains unaffected by serotonergic or noradrenergic drugs in vitro, highlighting its potential role in a pharmacoresistant mechanism [84]. The correlation of SNORA69 levels between LHb and peripheral blood in rodents highlight its potential to be used as a potential biomarker for depression which future studies should aim to recapitulate in human investigations [84].

## Habenular dysfunction in MDD: insights from human studies

### Molecular alteration associated with MDD

In line with findings from rodent models of depressive-like behaviors, qPCR analysis of the MHb of the postmortem human tissue revealed that CAPS2 mRNA levels in individuals with MDD were reduced to 73% of the levels observed in non-depressed controls [62]. Similarly, a study comparing postmortem human habenula tissue of those who died by suicide as compared to psychiatrically healthy controls reported significant downregulation of CHT and CHRNB3 mRNA levels, while other cholinergic genes (CHAT, VACHT, CHRNA3, and CHRNB4) exhibited non-significant decreases in expression [66]. In contrast, CRS rodent models show pronounced downregulation of both CAPS2 and cholinergic signaling transcripts [62, 66].

Studies have also aimed to investigate the role of Erb-B2 receptor tyrosine kinase 4 (ErbB4) in postmortem human tissue of individuals with MDD [87]. ErbB4 is a critical integration site for signaling processes and locates in the habenula [88–90]. The mRNA expression (qPCR) of ErbB4 has been found to be downregulated among the target genes of the differentially expressed microRNAs in the postmortem LHb of those with MDD [87]. More recent investigation using bulk small RNA sequencing in postmortem LHb tissue of individuals with MDD found that SNORA69 was also upregulated in postmortem LHb of individuals with MDD [84]. SNORA69, known to guide pseudouridylation onto 5.8S and 18S rRNAs, was found to have significantly elevated pseudouridine levels at both 5.8S and 18S ribosomal RNA (rRNA) sites in MDD, only the 18S rRNA modification correlated with SNORA69 expression [84]. Importantly, SNORA69 upregulation did not alter rRNA abundance, as evidenced by the lack of significant differences in these rRNA sites expression and the absence of correlation between SNORA69 levels and these rRNAs, suggesting its role in modifying ribosomal activity rather than rRNA stability [84].

Altogether, these findings underscore the critical role of molecular alterations in the postmortem habenula in the pathophysiology of MDD and highlight the need for translational studies that bridge controlled animal paradigms with the complexity of human depression. Future research should investigate how these changes interact to influence neural circuits and behavior, prioritizing mechanistic studies to determine how CAPS2 deficits, impaired cholinergic signaling (e.g., reduced CHT and CHRNB3), and SNORA69-mediated ribosomal pseudouridylation collectively disrupt habenular circuitry and drive depressive phenotypes [62, 66, 84]. Furthermore, in order to provide an unbiased and whole-transcriptome profile of habenular dysfunction in MDD, single-cell RNA sequencing could be used to resolve cell-type-specific molecular alterations, while spatial transcriptomic studies would help map localized changes in gene expression and synaptic signaling across neural circuits in habenular subregions.

### Macrostructural changes of habenular structure in MDD

In addition to molecular alterations, MDD pathology has also been linked with anatomical and functional changes to the habenula (Table 2). Due to the difficulty of distinguishing the neuroimaging signals from either the MHb or the LHb [91], relative heterogeneity in human subjects and lack of high throughput studies, investigations into macrostructural changes in the habenula seem to yield mixed findings [92, 93]. Indeed, functional magnetic resonance imaging (fMRI) studies of the whole habenula found a smaller average volume in those with MDD compared to healthy individuals, though this difference was not statistically significant [92]. Additionally, a negative association between habenula volume and anhedonia severity was found [92]. However, contradictory findings from another group showed significantly larger habenula volumes in those with MDD compared to healthy individuals, with a positive correlation between larger volumes and higher anhedonia severity [93]. These results showing larger habenula volumes do align with Schmidt et al., [94], who found that in unmedicated individuals with MDD, habenula volume was positively correlated with disease severity; specifically, individuals with moderate-to-severe MDD exhibited larger volumes than those with mild MDD. This suggests an early or acute increase in volume—a relationship that was not observed in medicated individuals with MDD when compared to non-depressed controls [94]. While the influence of sex on habenula volume in MDD remains unclear, some studies have found volumetric differences between females and males. An fMRI study of postmortem human habenula found that females with MDD had a lower total habenula volume than non-depressed controls, primarily due to a reduction in right habenula volume [95]. Interestingly, such differences in the total habenula volume was not observed in the male samples or in non-depressed controls [7, 91, 95, 96].

**Table 2:** Habenula volume of individuals with MDD compared to healthy individuals.

Postmortem Hb Samples			
	MDD volume loss compared to Healthy Individuals		Citation
	Left Side of Hb	Right Side of Hb	*Evidence for structural abnormalities of the human habenular complex in affective disorders but not in schizophrenia* [96] (*n* = 27; ~51.9% postmortem habenula from the individuals with MDD)
**Neuronal Cell Number**	−31.00%	−34.60%	
**Neuronal Cell Area**	−39.60%	−34.40%	
**MHb Volume**	−24.10%	−20.90%	
**LHb Volume**	−20.00%	−20.00%	
**fMRI of Unmedicated MDD Patient**			
	Left Side of Hb	Right Side of Hb	**Citations**
**Average Habenula volume**	−6.63%		*Disrupted habenula function in major depression* [92] (Siemens 3 T Magnetom TIM Trio Scanner; *n* = 50; 50% participants with MDD)
	Significantly Increased		*Association between habenula dysfunction and motivational symptoms in unmedicated MDD* [93] (*Philips 3* *T Achieva scanner;* *n* = 38; ~55.3% participants with MDD)

For postmortem samples [96], the Control group had a mean age of 55.9 ± 8.9 years, brain weight of 1322 ± 157 g, and postmortem delay of 28.6 ± 9.4 h; the MDD group had a mean age of 48.6 ± 12.4 years, brain weight of 1337 ± 133 g, and postmortem delay of 31.9 ± 24.8 h. For the first fMRI study [92], the mean age between the conditions is insignificant (Control: 27.44 ± 8.75 years and MDD: 27.76 ± 9.01 years, *p* = 0.90). For the second fMRI study [93], the mean age between the groups is insignificant (Control: 28.3 ± 5.2 years and MDD: 30.7 ± 8.9 years, *p* = 0.82).

*Hb* Habenula, *MDD* major depressive disorder, *MHb* medial habenula, *LHb* lateral habenula, *fMRI* functional magnetic resonance imaging.

While results from W.-H. Liu et al., [93] demonstrated an increase in habenula volume in individuals with MDD, it should be noted that some histological results have demonstrated a decrease in habenular volume. Indeed, a morphometric study of the habenula in postmortem tissue of individuals with MDD noted a significant decrease in both neuronal cell number and area (Table 2) [96]. Specifically, the right side of the habenula in individuals with MDD showed a 34.6% reduction in neuronal cell number and 39.6% in area, while the left side exhibited reductions of 31.0% in cell number and 34.4% in area, as compared to non-depressed controls [96]. Thus, this observed reduction in habenular volume in individuals with MDD, is potentially attributable to decreased neuronal number and reduced cell size as quantified by stereological analysis of serial histological sections. This reduction correlates with the severity of anhedonia, although interpretations of these findings vary within the scientific community.

Taken together, evidence suggests that structural alterations in the habenula are associated with MDD, however discrepancies exist in the directionality of the relationship; likely coming from methodological differences, potential sex-specific effects, and the influence of neuronal shrinkage that are discussed above. Future research should aim to refine imaging techniques to clearly differentiate the LHb from the MHb, integrate postmortem with in vivo neuroimaging findings, and increase both cohort size and imaging resolution. Such approaches will be critical in resolving the current contradictions and deepening our understanding of habenula’s role in the pathophysiology of depression.

### Alterations in functional connectivity patterns of the habenula in MDD

**Fig. 3:**
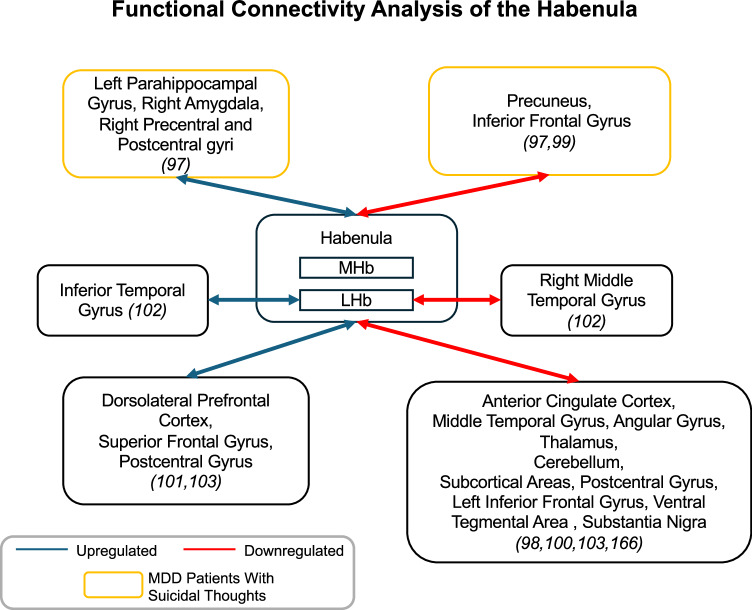
The functional connectivity (FC) analysis of the habenula in the development of MDD. The blue line indicates the upregulation of the functional connectivity between the habenula and this region, while the red line indicated the downregulation of the functional connectivity between the habenula and this region. The yellow box indicates the participants with MDD and suicidal thoughts. [102] was done on Siemens 3 T MRI system, and included 71 participants (*n* = 71; ~53.5% participant with MDD, ~60.6% female), with mean of age of 19.24 ± 0.94 years in control and of 21.13 ± 6.17 years in participants with MDD; [98] was done on Siemens 3 T MRI system, and included 84 participants (*n* = 84; ~54.77% participants with MDD, ~72.6% female), with mean of age of 37.1 ± 13.0 years in control and of 38.3 ± 12.5 years in participants with MDD; [100] was done on Philips Achieva 3 T MRI scanner, and included 50 participants (*n* = 50; 56% participants with MDD, 60% female), with mean of age of 39.0 (23.0–46.5) years in control and of 36.00 ± 10.37 years in participants with MDD; [103] was done on Philips Achieva 3 T MRI scanner, and included 100 participants (*n* = 100; 53% participants with MDD, 58% female), with mean of age of 28.94 ± 10.89 years in control and of 32.04 ± 10.01 years in participants with MDD; [101] was done on Siemens 3 T MRI system, and included 74 participants (*n* = 74; ~66.2% participants with MDD, ~59.5% female), with mean of age of 38.24 ± 10.14 years in control and of 34.80 ± 9.04 years in participants with MDD; [97] was done on 3 T Siemens Trio Magnetom system, and included 198 participants (*n* = 198; ~62.1% participants with MDD and suicide-related behaviours, ~52.5% female), with mean of age of 33.1 ± 9.1 years in control and of 29.7 ± 11.8 years in participants with MDD and suicide-related behaviours; [99] was done on 3 T Siemens Trio Magnetom system, and included 78 participants (*n* = 78; ~55.1% participants with MDD and suicidal ideation, ~53.8% female), with mean of age of 32.57 ± 8.75 years in control and of 33.12 ± 11.47 years in participants with MDD and suicidal ideation; and [165] was done in 7-Tesla resting-state fMRI subset of the WU-Minn HCP, and included 237 participants (*n* = 237; ~25.3% participants with MDD who received ketamine treatment, ~57.8% female), with mean of age of 29.4 ± 3.3 years in control and of 34.4 ± 11.5 years in participants with MDD who received ketamine treatment.

Recent fMRI work has reveal significant changes in the functional connectivity (FC) of the habenula in MDD, highlighting its key role as a communication hub in neural circuitry [97–103] (Fig. 3). Specifically, it has been noted that, as MDD symptoms progress, there is an observed increase in FC between the LHb and the inferior temporal gyrus, contrasted by a reduction in FC between the LHb and the right middle temporal gyrus [102]. One possibility is that, given the inferior temporal gyrus has been implicated in visual object recognition [104] and the right middle temporal gyrus is thought to mediate semantic memory and socio-emotional integration [105], altered FC between these cortical regions and the LHb might hypothetically enhance sensitivity to negative visual stimuli and impair the brain’s ability to contextualize emotional experiences, contributing to the anxiety or depressive phenotypes. Increased FC was also noted between the habenula and the dorsolateral prefrontal cortex (dlPFC) as well as the superior frontal gyrus in those with MDD [101, 103]. The dlPFC regulates executive functions such as decision-making, attention, and emotion regulation [106]. It is plausible that excessive coupling between the dlPFC and the habenula may overwhelm the dlPFC’s capacity to effectively modulate negative emotions, potentially contributing to persistent rumination or impaired reward-seeking behavior. Similarly, it is speculated that heightened connectivity between the habenula and the superior frontal gyrus [107], a region implicated in self-awareness [108], could amplify the examination of negative self-perceptions, potentially exacerbating feelings of guilt or worthlessness in individuals with MDD. Moreover, individuals with suicidal ideation, a hallmark feature of MDD, exhibit distinct FC profiles that are characterized by increased connectivity between the left habenula and regions such as the left parahippocampal gyrus, right amygdala, and right precentral and postcentral gyri [97].

On the other hand, diminished FC has been observed between the habenula and several brain regions, including the anterior cingulate cortex (ACC), middle temporal gyrus, angular gyrus, thalamus, cerebellum, subcortical areas, postcentral gyrus, and left inferior frontal gyrus [98, 100, 103]. These findings indicate a broad network disruption underlying the complex affective and cognitive impairments in depression. It is worth speculating that reduced connectivity with the ACC may foster hopelessness, as it is essential for emotion and conflict resolution [109]. Similarly, it is plausible that diminished thalamic connections may lead to mental fatigue and heightened stress sensitivity, given its role in sensory integration and arousal regulation [110]. Attenuated cerebellar links might compromise the fine-tuning of emotional responses, contributing to mood instability [111, 112]. Furthermore, it is speculated that disrupted integration with regions such as the angular gyrus and middle temporal gyrus might impaired delayed memory and contributing the severity of the MDD [113]. Diminished FC between the habenula and the precuneus and inferior frontal gyrus has also been observed in individuals with MDD and suicidal ideation compared to individuals with MDD without suicidal ideation and psychiatrically healthy controls [97, 99]. This reduced connectivity may reflect disruptions in neural circuits critical for integrating self-referential processing and behavioral regulation. The precuneus, a hub of the default mode network (DMN), supports self-awareness, autobiographical memory, and conscious reflection [114, 115]; weakened habenula-precuneus connectivity could potentially impair adaptive self-referential processing, potentially exacerbating rumination or feelings of hopelessness. The inferior frontal gyrus, involved in inhibitory control, emotion regulation, and decision-making [116–118], may fail to effectively manage emotional responses when communication with the habenula is impaired. These regions modulate emotional responses by regulating dopamine and serotonin release through the habenula, highlighting the key role of the habenula in regulating neural network dynamics and modulating neurotransmitter signaling.

Taking together, these findings may hint at the potential of leveraging altered fMRI FC of the habenula as biomarkers for early MDD diagnosis and even inform targeted therapies. Future studies should refine habenula connectivity mapping by employing advanced imaging techniques in larger, more diverse cohorts. Longitudinal research will be crucial to understand how fluctuations in connectivity correlate with symptom progression and treatment response. Moreover, integrating multimodal data, including molecular, genetic, and behavioral assessments, can further elucidate the complex interplay between the habenula and other brain regions, ultimately advancing therapeutic approaches for managing MDD.

## Potential therapeutic avenues for MDD targeting the habenula

### Proposed mechanism of action of ketamine in the NRG1-ErbB4 pathway in the habenula

As previously noted, ErbB4 is downregulated in the MHb of individuals with MDD, indicating its potential role in the underlying mechanisms of the disorder [87]. This receptor binds with high affinity to and is activated by neuregulin 1 (NRG1), a neurotrophic factor that regulates GABAergic transmission [119]. Both ErbB4 and NRG1 are localized in parvalbumin-positive (PV) neurons, which are fast-spiking interneurons in the LHb that are predominantly non-inhibitory, except within the LHb lateral subregion [120, 121]. Disruptions of these proteins in PV neurons have been implicated in the development of depression [87, 122–125].

**Fig. 4:**
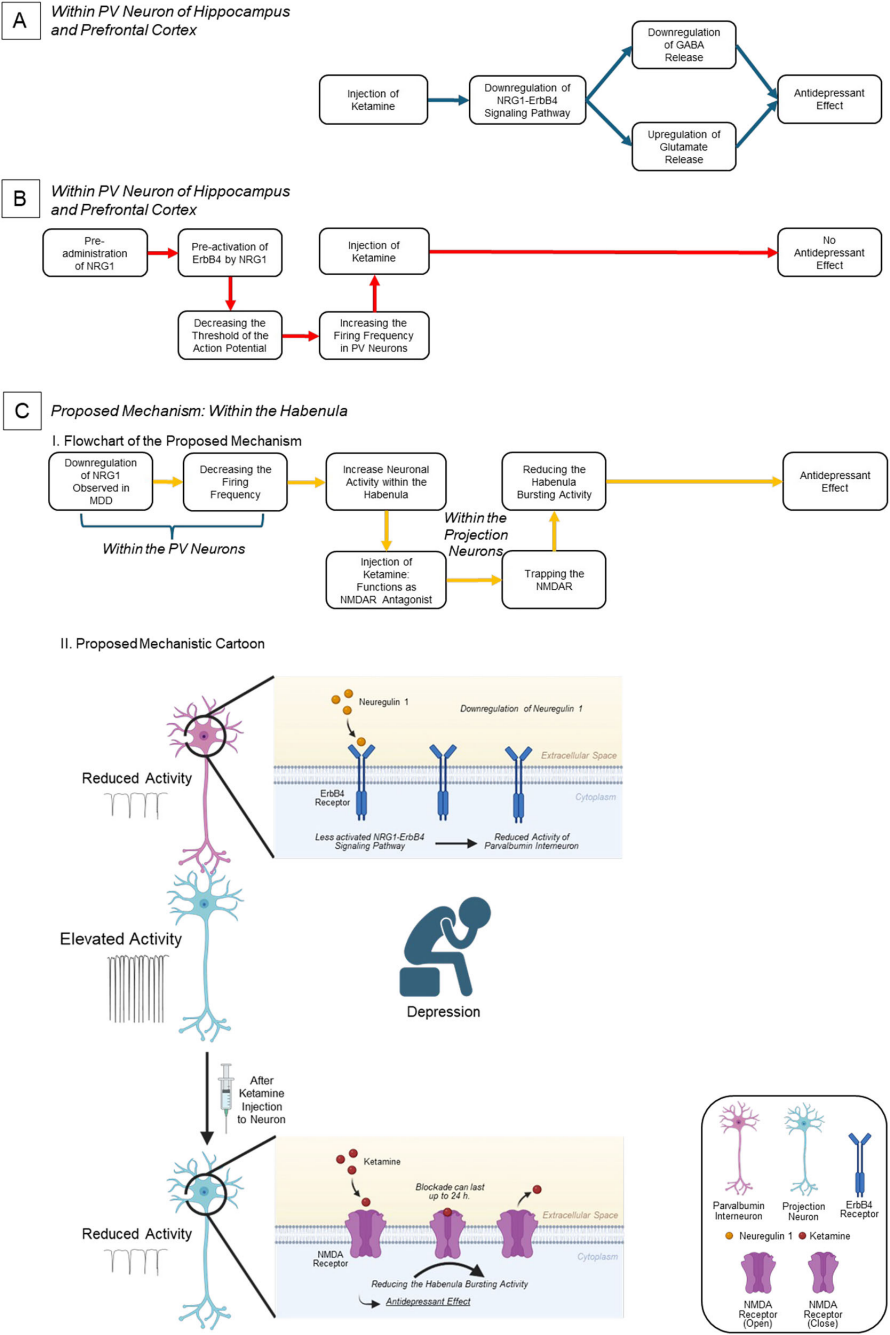
Schematic illustration of ketamine’s mechanism of action via the NRG1-ErbB4 pathway. **A** Administration of ketamine alone (without prior NRG1 treatment) within PV neurons in hippocampus and prefrontal cortex produces an antidepressant effect. **B** Pre-administration of NRG1 disrupts the antidepressant effect of ketamine within PV neurons in hippocampus and prefrontal cortex. **C** Proposed mechanism of ketamine’s antidepressant action in the habenula: Reduced NRG1 lowers ErbB4 signaling in PV neurons, allowing ketamine to more effectively suppress activity through NMDAR antagonism and trapping. C(I) Flowchart of the proposed mechanism; C(II) Receptor–ligand schematic.

The NRG1-ErbB4 signaling pathway is essential for neurotransmission and neuronal network synchronization by enhancing precisely timed GABA release, essential for emotion processing (Fig. 4) [126] and known to be implicated in the antidepressant effects of ketamine [127]. Ketamine is a NMDAR antagonist typically used as an anesthetic and has been shown to have significant antidepressant effects [128–131]. Ketamine administration in rodents’ hippocampus and prefrontal cortex has been shown to significantly downregulate NRG1, phosphorylation of ErbB4 (a sign of protein activation) within the PV neurons, downregulate GABA and upregulate glutamate [127]. However, pre-administration of NRG1 significantly reversed ketamine’s antidepressant effects, suggesting that it may be a rate limiting step in the antidepressant action of ketamine [127]. Furthermore, pre-activation of ErbB4 by NRG1 diminishes ketamine’s antidepressant effects and blocks downregulation of phosphorylation [127]. Recent work in the habenula demonstrated that ketamine blocks LHb bursting activity during MDD onset by trapping NMDA receptors in LHb neurons, as evidenced by the sustained blockade of NMDAR-mediated currents even after ketamine washout [132, 133]. Supporting this, research links NRG1 downregulation in the SD rodent models to the presence of depressive-like symptoms, albeit focusing on the medial prefrontal cortex neurons [134]. Putting together, we speculate that a similar mechanism may occur in the habenula, where reduced NRG1 expression leads to downregulation of NRG1-ErbB4 signaling in the PV neurons. Under these conditions, ketamine can more effectively suppress neuronal activity by acting as an NMDAR antagonist and trapping the receptor in the habenular projection neuron. However, future studies are needed to clarify this effect.

NMDAR plays an essential role in mediating bursting activities in the LHb neurons and blockade of NMDAR-mediated currents has been shown to rescue depressive symptoms, underscoring its therapeutic potential [132, 133]. Of particular relevance is the GluN2B subunit, a component of NMDAR critical for glutamate neurotransmission. This subunit is directly targeted by the NRG1-ErbB4 signaling pathway and contributes to processes central to MDD pathophysiology, such as neuroplasticity and emotion regulation [135–139]. Notably, GluN2B-containing NMDARs are present in the LHb and implicated in depressive symptoms, but the specific influence of the NRG1-ErbB4 signaling pathway on these receptors and how this may be linked to the mechanism of ketamine in the LHb remains poorly understood. Further investigation could integrate insights from studies in other brain regions. For instance, cortical research demonstrates that GluN2B phosphorylation at the Y1070 site correlates with depression severity and that ketamine’s antidepressant effects depend on GluN2B-mediated signaling [140, 141]. Building on this, experiments could test whether NRG1-ErbB4 activation in the LHb drives GluN2B phosphorylation at Y1070, thereby amplifying NMDAR activity and contributing to LHb hyperexcitability in MDD. Pharmacological or genetic inhibition of ErbB4 in preclinical models could clarify whether blocking this pathway reduces GluN2B phosphorylation, restores synaptic plasticity in the LHb, and alleviates depressive-like behaviors. Additionally, since ketamine’s cortical effects involve GluN2B inhibition and synaptic remodeling [140], studies should explore whether ketamine similarly modulates NRG1-ErbB4-GluN2B signaling in the LHb.

### Application of deep brain stimulation in treating MDD through LHb stimulation

Deep brain stimulation (DBS), has recently been used as a treatment option for those with MDD and involves the strategic placement of electrodes in specific brain regions to alleviate symptoms of MDD [142]. Interestingly, successful DBS implementation throughout the entire habenula has demonstrated efficacy in ameliorating depressive symptoms in individuals with treatment-resistant MDD [143–145]. Recent work has been done to understand why this DBS treatment in the habenula yielded such profound results. Specifically, Zhang et al., [146] demonstrated that DBS of the LHb alleviates depression-like behaviors in UCMS rodent models by normalizing neuronal hyperactivity and burst firing patterns [146]. Furthermore, measurements of pathway coherence revealed that DBS reduces connectivity between the LHb and the VTA, thereby restoring healthy dopaminergic and serotonergic signaling and shifting neural activity from burst-dominated to regular firing patterns [146].

It is important to note the influence of variables such as patient age and personalized DBS parameters tailored for individualized treatments. Despite successful clinical outcomes, DBS implementation raises critical questions that need further scientific inquiry. First, despite the successful implementation of DBS in MDD, the intricate mechanisms within the habenula, particularly focusing on the LHb, remain elusive. Therefore, a comprehensive understanding of these mechanisms needs further investigations employing cellular experiments and animal models to uncover the nuanced processes involved [147]. Second, challenges posed by the small size of the habenula within the brain, compounded by the difficulty in precisely targeting the LHb in DBS [95, 148]; underscoring the necessity for continuous optimization efforts. As the understanding of the habenula’s role in mental health disorders evolves, ongoing efforts in both experimental and clinical realms will contribute to refining DBS techniques and optimizing therapeutic outcomes for individuals with MDD.

## Habenular dysfunction in MDD comorbid disorders

Although this is not the focus of the review, it is important to note that mental health disorders that are highly co-morid with MDD also have habenula dysfunction in their pathology. For example, extensive evidence indicates a robust comorbidity between MDD and substance use disorders (SUD) [149–152]. Notably, the MHb is highly enriched with nicotinic acetylcholine receptors (nAChRs), which have been strongly implicated in SUD [153, 154]. To investigate the role of the habenula in alcohol use disorder, previous work began saccharin pairing with an intraperitoneal injection of ethanol to induce conditioned taste aversion (CTA) [155]. In vivo electrophysiological recordings revealed that LHb neurons of the rats (*n* = 6) exhibited significantly increased baseline and stimulus-evoked firing during an operant task for saccharin compared to recordings made without ethanol exposure [155]. This heightened LHb activity is thought to mediate ethanol-induced aversion via its excitatory projections to inhibitory neurons in the RMTg, which suppress dopamine release from VTA neurons, a key process underlying reward-seeking behavior [33, 155]. In addition, the LHb may influence CTA through the dorsal raphe pathway, where altered serotonergic signaling contributes to encoding updated reward values [155]. To assess the necessity of LHb activity in the behavioral expression of ethanol-induced CTA, two groups of rats were compared: sham-operated rats and those with bilateral LHb lesions. The LHb-lesioned rats exhibited markedly attenuated behavioral aversion while demonstrating unchanged licking frequencies during saccharin consumption, indicating that the increased LHb firing is crucial for suppressing reward-seeking behaviors in ethanol-induced conditioned taste aversion [155]. Similarly, in vitro electrophysiological recordings of LHb neurons in rodent brain slices showed that the application of cocaine depolarized neurons and significantly increased both spontaneous firing and evoked glutamatergic postsynaptic currents [156]. In parallel, in vivo rodent extracellular recordings from LHb neurons demonstrated that intravenous cocaine initially suppressed LHb firing during its rewarding phase, but this inhibition was followed by a delayed rebound excitation that paralleled the emergence of aversive conditioning. This delayed activation was further confirmed by increased c-Fos immunoreactivity, reflecting reduced downstream dopamine signaling [157]. Moreover, in experiments conducted on mice, repeated cocaine administration over two consecutive days led to long-term hyperactivity in LHb neurons by enhancing glutamatergic transmission, as demonstrated by increased AMPA receptor-mediated excitatory currents, altered AMPA/NMDA ratios, and glutamate uncaging [158]. In a nicotine addiction study using brain slices from the rodents, including both control and α6-nAChR knockout, nicotine was applied at concentrations ranging from low nanomolar to micromolar levels that mimicking those found in human nicotine smokers to assess its effects on LHb neurons using electrophysiological recording [159]. The results showed a biphasic response, with an initial transient decrease in neuronal firing due to enhanced GABAergic signaling via α4β2 receptors followed by a sustained increase in firing mediated by increased glutamate release through α6-containing receptors [159]. This pattern suggests that the LHb plays a critical role in regulating the motivational properties of nicotine by modulating both its aversive and rewarding effects, which are fundamental to nicotine addiction.

The MHb, which contains high densities of nAChRs, particularly α3, α5, and β3 subunits, is strongly implicated in nicotine addiction [153, 154]. Fowler et al., [160] demonstrated that α5-containing nicotinic receptors, particularly those in the MHb, play a crucial role in controlling nicotine intake. Indeed, mice lacking the α5 subunit exhibited significantly higher nicotine consumption, especially at elevated doses. When the α5 subunit was reintroduced specifically into the MHb, nicotine intake was normalized, underscoring the pivotal role of MHb nAChRs in mediating an inhibitory signal that limits drug consumption [160]. Complementary experiments in rats, using lentiviral knockdown of α5 expression in the MHb, yielded similar increases in nicotine intake and blunted responses in downstream brain regions such as the IPN [160]. Furthermore, blocking nAChRs in the MHb with the antagonists mecamylamine induces withdrawal-like symptoms in nicotine-dependent rodents, emphasizing its involvement in withdrawal regulation [161].

## Conclusion

In this review, we provide a comprehensive overview of the habenula’s role in regulating brainstem neuromodulatory systems, highly implicated dysfunction in MDD, and current research regarding potential therapeutics for MDD that target the habenula. Our review builds upon several recent comprehensive reviews [4, 162–164], by integrating and extending their insights into how habenular dysfunction contributes to depression. The novelty of this review lies in its comprehensive and in-depth exploration of the molecular mechanisms underlying habenula dysfunction in MDD. Indeed, in our review, we summarized the most recent work on rodent models, including UCMS, CRS, and LH paradigms, which have revealed key molecular alterations in the habenula linked to MDD. These alterations include the downregulation of CAPS and cholinergic gene expression in the MHb [62, 66], upregulation of p11, REM sleep-active neurons, stress-responsive neurons, TCF7L2, Kir4.1, βCaMKII, and PP2A, along with SNORA69, in the LHb [69, 72, 74, 76, 78–80]. Similar molecular changes of CAPS2, cholinergic signalling, p11, SNORA69 and ErbB4 [62, 66, 76, 84, 87] were also observed in the postmortem habenula of the individual with MDD [62, 66, 76, 84, 87]. Clinical studies of MDD have also observed macrostructural alterations of the habenula, including volumetric changes [92–94], decreased neuronal size and number [96], along with disrupted functional connectivity to other brain regions [97–103]. We also describe potential therapeutic interventions like ketamine and DBS which offer promising antidepressant effects and seem to exhibit these effects through habenular manipulation [142, 147]. To advance this field, future research should prioritize integrative approaches, such as single-cell sequencing and spatial transcriptomics, to resolve cellular heterogeneity across habenular subregions and delineate precise molecular pathways driving MDD. Such efforts will accelerate the identification of novel biomarkers and the development of targeted therapies for MDD through habenula-specific interventions.
